# Therapeutic monoclonal antibody for sporotrichosis

**DOI:** 10.3389/fmicb.2012.00409

**Published:** 2012-11-28

**Authors:** Sandro R. Almeida

**Affiliations:** Faculty of Pharmaceutical Sciences, Department of Clinical e Toxicological Analysis, University of São PauloSão Paulo, Brazil

**Keywords:** fungal infection, immunology, medical mycology, monoclonal antibody, sporothrix, sporotrichosis, vaccine, yeast

## Abstract

Sporotrichosis is a chronic subcutaneous mycosis that affects both humans and animals worldwide. This subcutaneous mycosis had been attributed to a single etiological agent, *Sporothrix schenckii*. *S. schenckii* exhibits considerable genetic variability, and recently, it was suggested that this taxon consists of a complex of species. Sporotrichosis is caused by traumatic inoculation of the fungus, which is a ubiquitous environmental saprophyte that can be isolated from soil and plant debris. The infection is limited to cutaneous forms, but recently, more severe clinical forms of this mycosis have been described, especially among immunocompromised individuals. The immunological mechanisms involved in the prevention and control of sporotrichosis are not well understood. Some studies suggest that cell-mediated immunity plays an important role in protecting the host against *S. schenckii*. In contrast, the role of the humoral immune response in protection against this fungus has not been studied in detail. In a previous study, we showed that antigens secreted by *S. schenckii* induced a specific humoral response in infected animals, primarily against a 70-kDa molecule, indicating a possible role of specific antibodies against this molecule in infection control. In another study by our group, we produced a mAb against a 70-kDa glycoprotein of *S. schenckii* to better understand the effect of the passive immunization of mice infected with *S. schenckii*. The results showed a significant reduction in the number of CFUs in various mice organs when the mAb was injected before or during *S. schenckii* infection. Similar results were observed when T-cell-deficient mice were used. The drugs of choice in the treatment of sporotrichosis require long periods, and relapses are frequently observed, primarily in immunocompromised patients. The strong protection induced by the mAb against a 70-kDa glycoprotein makes it a strong candidate as a therapeutic vaccine against sporotrichosis.

## Introduction

Sporotrichosis is a chronic fungal infection that is endemic to Brazil, and it is the most common subcutaneous mycosis in South America (Schubach et al., 2008). The disease is mainly caused by the dimorphic fungus *Sporothrix schenckii*. In its saprophytic stage or when cultured at 25°C, it assumes a filamentous form, and at 37°C, it assumes a yeast form (Bustamante and Campos, 2001; Barros et al., 2011). *Sporothrix* is widely distributed in nature and exists in a saprophytic mycelial form in plant debris and soil. The traumatic inoculation of the conidia and hyphae of this fungus results in the development of subcutaneous mycoses; within the infected tissue, the fungus differentiates into its yeast form and may spread to other tissues (Ramos-e-Silva et al., 2007; Barros et al., 2011). Since the 1980s, domestic cats have been a source of mycosis transmission to humans (Nusbaum et al., 1983; Dunstan et al., 1986a,b; Larsson et al., 1989; Fleury et al., 2001). The largest epidemic of sporotrichosis due to zoonotic transmission was described in Rio de Janeiro between 1998 and 2004, in which 759 humans were diagnosed with sporotrichosis (Barros et al., 2004; Freitas et al., 2010). Recently, Marimon et al. (2007) suggested that *S. schenckii* should not be considered the only species that causes sporotrichosis on the basis of a combination of phenotypic and genetic features. The group described four new species: *S. globosa*, *S. brasiliensis*, *S. mexicana*, and *S. luriei* (Marimon et al., 2008). These new species have been defined as having a worldwide distribution, whereas *S. brasiliensis* is apparently restricted to Brazil, and *S. mexicana* is restricted to Mexico. Due to difficulties in classifying strains belonging to the *Sporothrix* complex, the same group (Marimon et al., 2008) proposed an identification key that includes the analysis of conidial morphology, auxanogram analysis using raffinose and sucrose and genotyping via polymerase chain reaction (PCR) amplification of the calmodulin gene.

Sporotrichosis has diverse clinical manifestations. The most frequent clinical form (approximately 80% of cases) is the lymphocutaneous form (Bonifaz and Vazquez-Gonzalez, 2010). It starts with a nodular or ulcerated lesion at the site of fungal inoculation and follows a regional lymphatic trajectory characterized by nodular lesions that ulcerate, fistulate, and heal, representing true gummae. Another common clinical manifestation is the fixed cutaneous form. In general, the fixed cutaneous form is characterized by infiltrated nodular, ulcerated, or erythematosquamous lesions located on exposed areas on which the fungal inoculation occurred (Schechtman, 2010). The systemic form of sporotrichosis may evolve from an initial cutaneous lesion or be associated with the inhalation of conidia (Gutierrez-Galhardo et al., 2010). More severe clinical forms of this disease have been associated with immunocompromised patients, such as human immunodeficiency virus (HIV)-infected patients, suggesting that *S. schenckii* is an emerging opportunistic pathogen (Galhardo et al., 2010). In contrast, disseminated cutaneous sporotrichosis has been reported in an immunocompetent individual (Yap, 2011), which demonstrates that although it is common in immunosuppressed patients, disseminated cutaneous sporotrichosis can also present in immunocompetent patients.

Different drug protocols are used for the treatment of sporotrichosis, including potassium iodide, itraconazole, terbinafine, fluconazole, and amphotericin B (Song et al., 2011). The treatment choice is based on the individual's clinical condition, the extent of the cutaneous lesions, the assessment of drug interactions and adverse events, and systemic involvement. Some adverse events, such as nausea, vomiting and diarrhea, headache, abdominal pain, hypersensitivity reactions, and liver dysfunction, may be observed (Lopez-Romero et al., 2011).

Both the pathogenic characteristics of the individual *S. schenckii* strains and the immunologic status of the host can determine the clinical manifestations of sporotrichosis. However, the factors involved in the pathogenesis of *S. schenckii* and mechanisms to determine the strain's virulence remain unclear.

## Immune response

The host defense against pathogenic microorganisms includes innate and acquired immunity. The innate immune system is the first line of defense against invading pathogens; it is activated within minutes after the invasion of the host and is responsible for defense during the initial hours and days of the infection. In contrast to the rapid activation of the innate immune system, the activation of specific acquired immunity, which is primarily mediated by T and B lymphocytes, requires at least 7–10 days before a proper cellular or humoral response occurs. The ability to discriminate “non-self” from “self” is an essential component of innate immunity and is achieved through germ line-encoded receptors that recognize highly conserved microbial structures: pathogen-associated molecular patterns (PAMPs).

Several classes of recognition receptors mediate the recognition of fungal pathogens. Because of the structure of the fungal cell wall, which is mainly composed of carbohydrate chains, such as mannans and glucans, the first group of fungal recognition receptors discovered was the lectin receptors (Brown, 2008; Netea et al., 2008). PAMPs are recognized by pattern recognition receptors (PRRs), which are expressed on various antigen-presenting cells (APCs), such as dendritic cells (DC) and macrophages. PRRs play an important role in recognizing microbial pathogens, activating the innate immune system, and releasing pro-inflammatory cytokines.

Recent studies have demonstrated that lipid extracts from the yeast form of *S. schenckii* bind to TLR4, and this interaction leads to the induction of an oxidative burst against the fungus (Sassa et al., 2012).

Uenotsuchi et al. (2006) showed that *S. schenckii* of cutaneous origin but not visceral origin binds to TLR2 and/or TLR4 and induces JNK, ERK, and p38 MAPK activation and IL-6 and TNF-α release. Although both TLR2 and TLR4 ligation can induce these pro-inflammatory cytokines, TLR2 signals are also known to mediate an anti-inflammatory effect by directing the release of IL-10. Thus, the authors speculate that TLR4 but not TLR2 might be the main receptor for recognizing *S. schenckii* of cutaneous origin and inducing a strong Th1 immune response.

T cell-mediated immunity has been described as being fundamental in the defense against *S. schenckii*. In experimental infections, nude mice are more susceptible to sporotrichosis, and acquired immunity against *S. schenckii* is primarily mediated by macrophages that have been activated by T cells (Hachisuka and Sasai, 1981). Both CD4^+^ T cells and macrophages are required in the granulomatous skin lesions of sporotrichosis; the presence of IFN-γ-producing CD4^+^ T cells is an essential component of host defense against this pathogen (Tachibana et al., 1999). These findings indicate that the Th1 response mediates granuloma formation in sporotrichosis.

Cell-mediated and innate immunity are considered the most important mechanisms of host defense against fungal infections. In contrast, the role of the humoral immune response in protection against this fungus has not been studied in detail. Recent studies have demonstrated that antibodies with defined specificity show different degrees of protection against mycosis (Casadevall and Pirofski, 2005). The administration of mAbs-mediated protection against *Paracoccidioides brasiliensis* (Buissa-Filho et al., 2008), *Candida albicans* (Polonelli et al., 2003), *Histoplasma capsulatum* (Nosanchuk et al., 2003) and *Cryptococcus neoformans* (Rivera et al., 2005) infection in mice.

## Therapeutic monoclonal antibodies and fungal infection

Monoclonal antibodies are attractive biologic drugs because of their specificity and well-understood mechanisms of action, which result in a higher predictability and lower attrition rate compared with other drugs. The mechanisms of antibody action against infectious disease include complement-mediated lysis, the enhancement or inhibition of phagocytosis, Fc-mediated cytokine release, and direct antimicrobial effects (Casadevall and Pirofski, 2005). In terms of fungal infection, it is clear that antibody-mediated immunity can be decisive for host defense against *C. neoformans* (Casadevall and Pirofski, 2012). However, several factors could affect the function of these antibodies, such as antibody isotype and quantity.

For many years, the protective role of antibodies in fungal infection was contested. A consensus has now emerged that the inability of immune sera to mediate protection against fungi reflects inadequate amounts of protective antibody and/or the simultaneous presence of protective and non-protective antibodies rather than a fundamental inability of antibodies to protect against fungal pathogens. Recently, several studies have established that some antibodies are protective against fungi (Xander et al., 2007; Buissa-Filho et al., 2008; Toledo et al., 2010; Zhang et al., 2011). Moreover, some MAbs designed to protect against cryptococcosis (Larsen et al., 2005) and candidiasis (Pachl et al., 2006) have been approved for clinical evaluation. An HIV+ patient with cryptococcal meningitis was treated with the murine MAb 18B7, which is directed against the capsular polysaccharide of *C. neoformans*, with excellent results. The MAb infusion had a half-life in the serum of approximately 53 h and reduced the fungal circulating antigen (Larsen et al., 2005). The phase I trial of Mab 18B7 has been completed. Additional phase II and III studies will be required to demonstrate whether adjunctive therapy with anti-*Cryptococcus* antibody confers additional benefit over conventional antifungal therapy. In another study, a human recombinant MAb to heat shock protein 90 was used in the treatment of patients with invasive candidiasis (Pachl et al., 2006). Recently, Krenova et al. (2010) reported the successful treatment of life-threatening *Candida peritonitis* in a child with abdominal non-Hodgkin lymphoma using a MAb against heat shock protein 90 in association with amphotericin B.

In a previous study, we demonstrated that mice infected with *S. schenckii* are able to produce specific IgG1 and IgG3 antibodies against a 70-kDa fungal protein during experimental infection, indicating that specific antibodies against this molecule may participate in controlling infection (Nascimento and Almeida, 2005). To better understand the role of the antibody response in sporotrichosis, our group produced an IgG1 mAb, P6E7, against a 70-kDa glycoprotein (gp70) of *S. schenckii*. Immunolocalization using this anti-gp70 antibody showed that the antigen was preferentially localized on the cell surface and that it could be a putative adhesin for fibronectin and laminin. To analyze the protective effect of the mAb *in vivo*, *S. schenckii*-infected mice were passively immunized. Our results showed a significant reduction in the number of CFUs in the spleen and liver of mice when the mAb was injected before and during *S. schenckii* infection (Nascimento et al., 2008). Furthermore, in a second experiment, the mAb was injected after infection was established, and again, we observed a significant reduction in CFUs. IFN-γ was detected at high levels in the organs of mice that received P6E7. These results indicate that treatment with the P6E7 mAb may induce a protective cell-mediated immune response via the production of IFN-γ (Nascimento et al., 2008). In a recent study, our group showed that yeast cells opsonized with mAbs against gp70 had an increased phagocytic index and TNF-α production (Franco et al., 2012).

A possible limitation to the use of vaccines in immunosuppressed patients is that these patients may not mount protective responses, but passive immunization with protective antibodies may well be a rapid and effective preventive or even therapeutic measure. The efficacy of this immunoprophylaxis can be augmented when it is used in combination with conventional antifungal therapy. Because the disseminated cutaneous forms of sporotrichosis have mainly been observed among immunosuppressed patients, especially HIV+ individuals, we analyzed the protective effect of the anti-gp70 mAb in deficient nude animals. We demonstrated that in this model, the mAb was efficient at controlling the dissemination of fungal infection (Nascimento et al., 2008).

The results of these studies may facilitate the development of an efficient therapy for sporotrichosis. Currently, our laboratory is in the process of developing a humanized P6E7 mAb. The humanized antibodies could be an alternative therapy for the treatment of patients with sporotrichosis.

## Conclusion

Therapeutic monoclonal antibodies, which are primarily used as treatment for cancer and less frequently for infection, are among the most active area of research and development in the pharmaceutical industry. mAbs can be used as conjugates with target drugs and can be fragmented or humanized. A better understanding of the mechanism of action of successful mAbs will be critical for the development of more active and less toxic mAbs.

The development of a therapeutic vaccine that has the ability to induce a strong protective response to *S. schenckii* could therefore be more advantageous than existing treatments. The development of a new mAb-based therapy is an exciting challenge that may lead to novel approaches in the treatment and immunoprophylaxis of sporotrichosis.

### Conflict of interest statement

The author declares that the research was conducted in the absence of any commercial or financial relationships that could be construed as a potential conflict of interest.
